# Skull Base Osteomyelitis in the Emergency Department: A Case Report

**DOI:** 10.1155/2011/947327

**Published:** 2011-05-29

**Authors:** Mustafa Burak Sayhan, Cemil Kavalci, Ozgur Sogüt, Eylem Sezenler

**Affiliations:** ^1^Department of Emergency Medicine, Faculty of Medicine, Trakya University, Edirne 22030, Turkey; ^2^Department of Emergency Medicine, Faculty of Medicine, Harran University, Sanliurfa 63000, Turkey

## Abstract

Skull base osteomyelitis (SBO) is a rare clinical presentation and usually occurs as a complication of trauma or sinusitis. A 5-year-old child presented to the emergency department with a three-week history of fever associated with drowsiness and left parietal headache, and a week's history of swelling on the left frontoparietal soft tissue. He had suffered a penetrating scalp injury four month ago. On physical examination, there was a tender swelling with purulent stream on the lateral half of his scalp. His vital signs are within normal limits. Plain X-ray of the skull showed a lytic lesion on the left frontoparietal bone. A cranial computed tomography (CT) scan demonstrated a large subgaleal abscess at the left frontoparietal region. SBO possesses a high morbidity and mortality; therefore, prompt diagnosis and appropriate treatment are mandatory to prevent further complications and to reduce morbidity and mortality significantly.

## 1. Introduction

Osteomyelitis can affect any bone. The common sites are the long bones especially the tibia and fibula. Skull base osteomyelitis (SBO) is a rare clinical presentation [1]. Osteomyelitis of the frontal bone associated with subperiosteal abscess collection is termed Pott's puffy tumour [2]. Sir Percivall Pott described Pott's puffy tumour in 1768 as a local subperiosteal abscess and osteomyelitis of the frontal bone resulting from trauma [3]. The prevalence of SBO is about 1.5% of all osteomyelitis [1]. The overall incidence of skull base osteomyelitis ranged from 57 to 95 cases annually [4]. 

SBO, which is a true bony infection, originates mostly from a chronic infection, which has been inadequately treated [5]. It can affect the calvarium or the base of the skull. In children trauma is the commonest predisposing factor [1]. Etiology of SBO may result from trauma, bone surgery, bacteremia, or a contiguous infectious focus and is further influenced by various diseases which affect the vascularity of bone, as well as by systemic diseases that can produce an alteration of host defenses. Radiation, malignancy, osteoporosis, osteopetrosis, and Paget's disease are all conditions that decrease the vascularity of bone and, therefore, cause a predisposition to infection [1, 4, 5]. Mortality from complications in SBO is 20–40%. Early diagnosis and appropriate management of SBO can prevent neurologic deficits and reduce morbidity and mortality significantly [1]. We described such a rare case in a 5-year-old child presenting with skull base osteomyelitis secondary to penetrating scalp injury.

## 2. Case Report

**Figure 1:**
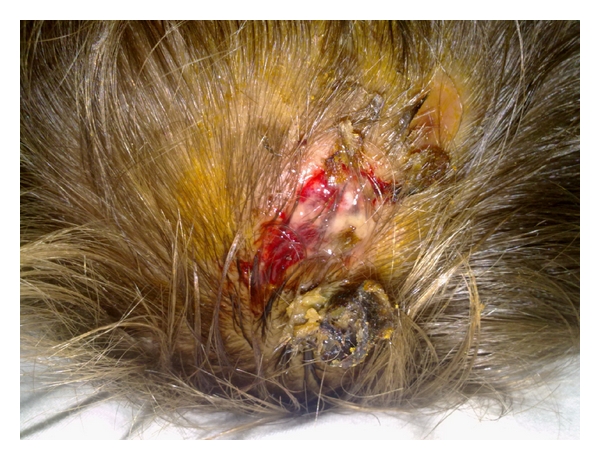
A view of purulent stream and tender swelling on the patient's lateral half of scalp.

**Figure 2:**
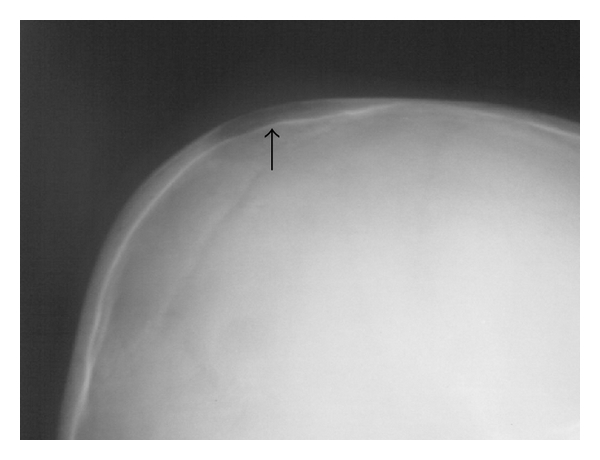
Plain radiograph of the skull taken at time of presentation showing a lytic lesion on the left frontoparietal bone.

**Figure 3:**
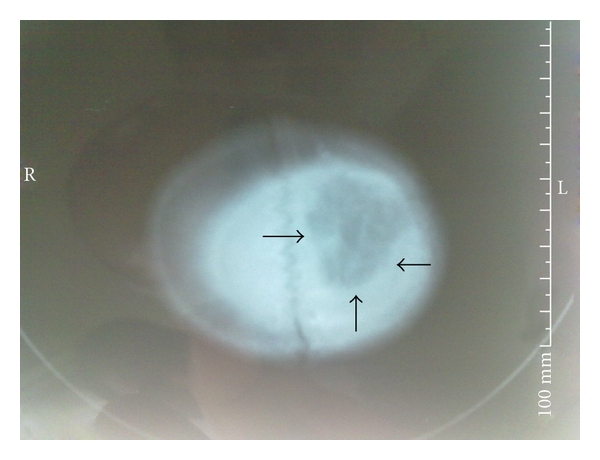
Axial computed tomography scan of the head (taken at time of presentation) through the left frontoparietal region shows a large subgaleal abscess.

A 5-year-old child presented to the emergency department (ED) on July 2, 2010 because of a three-week history of fever associated with drowsiness, and left parietal headache, and a week's history of swelling on the left frontoparietal soft tissue. The patient had suffered a penetrating scalp injury resulting from motor vehicle-pedestrian accident four month ago. He had never admitted to the hospital nor received a medical treatment. There was no previous history of any other concurrent medical conditions. On physical examination, there was a tender swelling associated with purulent stream on the lateral half of his scalp (Figure 1). Initial ED evaluation revealed a hemodynamically stable patient with an oral temperature of 38.9°C, a blood pressure of 120/85 mmHg, a heart rate of 96 beats/min, and a respiratory rate of 17 breaths/min. He had minimal neck stiffness but, Brudzinski's and Kernig's signs were negative. Furthermore, his pupils were equal in size and reactive. Examination of his respiratory, abdominal, and cardiovascular systems were normal. Plain X-ray of the skull showed a lytic lesion on the left fronto-parietal bone (Figure 2). A cranial computed tomography (CT) scan demonstrated a large subgaleal abscess at the left frontoparietal region (Figure 3). The leukocyte count, erythrocyte sedimentation rate and C-reactive protein (CRP) were 24900/uL, 40 mm/h, and 5.7 mg/dl, respectively. Other laboratory studies including blood chemistry and urine analysis were within normal ranges. He was started on intravenous ceftriaxone and clindamysin. By day 7 the treatment with oral amoxicillin/clavulanic acid (45 mg/kg/day divided every 12 hours) for 10 days was continued. Debridement of infected soft tissues and bone was performed by a neurosurgeon. The patient was discharged from the hospital on the 14th day following admission with no residual neurologic deficits, to be followed up in neurosurgery outpatient clinic. 

## 3. Discussion

Osteomyelitis of skull bones is uncommon particularly in children. It can affect the calvarium or the base of the skull [1]. Anatomically, the bones involved in osteomyelitis of the skull include the mandible, frontal bone, maxilla, nasal bone, temporal bone, and skull base bones [4]. In the present case, left frontoparietal region of the skull was affected. 

Etiology may result from trauma, bone surgery, bacteremia, or a contiguous infectious focus and is further influenced by diseases that affect the vascularity of bone, as well as by systemic diseases that produce an alteration of host defenses. Systemic diseases that reduce host defenses include diabetes, anemia, radiation, malignancy, and malnutrition [3, 4]. Most cases of skull osteomyelitis are related to trauma [6]. In the present case, there was no prior history of bone surgery to the face or no comorbid diseases except malnutrition, but he had been sustained a penetrating scalp injury four month ago. He had never admitted to the hospital or received any treatment. 

Acute osteomyelitis may present as a routine infection with several signs including fever, malaise, pain, and facial cellulites [7]. The main clinical findings include headache, sometimes associated with edema and spontaneous drainage if a sinocutaneous fistula has formed [8]. There may not be any associated noticeable radiographic changes [9]. Radiologic diagnosis of skull base osteomyelitis should be fast and accurate [10]. A cranial computed tomography (CT) and magnetic resonance imaging (MRI) can be used for early detection [4]. Early features are seen as islands of normal bone with increased or diminished density. Advanced features are seen as lytic lesions [1]. It may take up to 10 to 12 days for bone loss to be apparent radiographically [6]. CT scan shows contrast-enhancing rim with a non-enhancing hypodense center [1, 6]. A cranial CT scan combines X-ray images taken from many different angles, creating detailed cross-sectional views of a person's internal structures [11]. In the present case, lytic lesions in the left frontoparietal bones at plain X-ray of the skull and a large subgaleal abscess at CT scan of the skull were demonstrated.

Useful laboratory values include elevated white blood cell count, certainly in the acute stages. Elevated erythrocyte sedimentation rate (ESR) and elevated C-reactive protein (CRP) may also be useful markers in both the diagnosis and treatment of osteomyelitis [4]. Monitoring of the ESR or CRP is one of the key investigations that can help to guide how long antibiotic therapy is continued, and its normalization would appear to be a good indicator that the infection has resolved [12]. In the present case, body temperature fell after the start of parenteral antibiotic therapy and debridement of involved soft tissue. Also, the clinical course in the present case was correlated with ESR and CRP levels. The patient responded quite well to the therapy of broad-spectrum antibiotics and surgical debridement with decreased activity levels in both ESR and CRP.

Acute osteomyelitis may be primarily managed with antibiotics [9]. Before the era of systemic antimicrobial therapy, skull base osteomyelitis was almost universally fatal [10, 12]. Broad-spectrum antibiotics are strongly recommended because the sites of primary infections vary and many different organisms can be the cause of the abscess formation. Brain abscess is the commonest complication of skull osteomyelitis. This is usually associated with subperiosteal abscess. The source of the infection must be eradicated [1]. Surgical treatment is usually focused on debridement of involved soft tissue and bone. Delay in surgical intervention has been associated with prolonged hospitalization [13]. Due to the implementation of effective antibiotics and early surgical intervention, the patient was discharged from hospital with no residual neurological problems.

## 4. Conclusion

Skull base osteomyelitis is a rare condition in children that usually require prompt diagnosis and treatment to avoid neurologic deficits and permanent disability and to reduce mortality. A combination of effective surgical debridement with prolonged appropriate antibiotic therapy in early term of skull base osteomyelitis might provide a complete resolution in all cases. Plain X-ray of the skull is helpful in establishing a diagnosis of osteomyelitis, but cranial CT is even more useful for determining the extent of the abscess.
